# Curcumin Nanoformulations for Colorectal Cancer: A Review

**DOI:** 10.3389/fphar.2019.00152

**Published:** 2019-03-05

**Authors:** Kar En Wong, Siew Ching Ngai, Kok-Gan Chan, Learn-Han Lee, Bey-Hing Goh, Lay-Hong Chuah

**Affiliations:** ^1^Biofunctional Molecule Exploratory Research Group, School of Pharmacy, Monash University Malaysia, Bandar Sunway, Malaysia; ^2^Novel Bacteria and Drug Discovery Research Group, Microbiome and Bioresource Research Strength, Jeffrey Cheah School of Medicine and Health Sciences, Monash University Malaysia, Bandar Sunway, Malaysia; ^3^Faculty of Science, School of Biosciences, University of Nottingham Malaysia, Semenyih, Malaysia; ^4^Division of Genetics and Molecular Biology, Institute of Biological Sciences, Faculty of Science, University of Malaya, Kuala Lumpur, Malaysia; ^5^International Genome Centre, Jiangsu University, Zhenjiang, China; ^6^Centre of Health Outcomes Research and Therapeutic Safety, School of Pharmaceutical Sciences, University of Phayao, Phayao, Thailand; ^7^Advanced Engineering Platform, Monash University Malaysia, Bandar Sunway, Malaysia

**Keywords:** colorectal cancer, colon cancer, curcumin, nanoformulations, nanoparticles, liposomes, micelles, nanogels

## Abstract

Colorectal cancer (CRC) is the third most prevalent form of cancer, after lung cancer and breast cancer, with the second highest death incidence. Over the years, natural compounds have been explored as an alternative to conventional cancer therapies such as surgery, radiotherapy, and chemotherapy. Curcumin, an active constituent of turmeric has been associated with various health benefits. It has gained much attention as an anticancer agent due to its ability to regulate multiple cell signaling pathways, including NF-κB, STAT3, activated protein-1 (AP-1), epidermal growth response-1 (Egr-1), and p53, which are crucial in cancer development and progression. Nevertheless, the clinical application of curcumin is greatly restricted because of its low water solubility, poor oral absorption, and rapid metabolism. These issues have led to the development of curcumin nanoformulations to overcome the limitations of the compound. Nanotechnology-based delivery systems have been widely used in improving the delivery of poorly-water soluble drugs. Besides, these systems also come with the added benefits of possible cellular targeting and improvement in cellular uptake. An ideal improved formulation should display a greater anticancer activity compared to free curcumin, and at the same time be non-toxic to the normal cells. In this review, we focus on the design and development of various nanoformulations to deliver curcumin for use in CRC such as liposomes, micelles, polymer nanoparticles, nanogels, cyclodextrin complexes, solid lipid nanoparticles (SLN), phytosomes, and gold nanoparticles. We also discuss the current pre-clinical and clinical evidences of curcumin nanoformulations in CRC therapy, analyse the research gap, and address the future direction of this research area.

## Introduction

Colorectal cancer (CRC) is the third most common cancer in the world, ranked after lung and breast cancer; and is the second most common cause of cancer death (WHO, 2018). Approximately 1.4 million people have newly identified CRC in 2012 alone (World Cancer Research Fund International, 2015). Conventional treatment options for cancer include chemotherapy, radiation, and surgery. Chemotherapy and radiotherapy are often associated with serious side effects and toxicity, thus significantly affecting patients' quality of life. Cancer cells have also been found to be able to develop resistance toward chemotherapy and radiotherapy over time (Yallapu et al., 2013). Overcoming the challenges in cancer treatment is crucial for better patient outcomes. This has led scientists to look for newer, alternative treatments. Lately, the use of natural compounds such as curcumin (CUR), resveratrol, lycopene, gingerol, and folate has gained much attention as alternatives to conventional therapies. These natural compounds have been shown to possess chemopreventive and/or anticancer activities with minimal side effects (Guilford and Pezzuto, 2008). Over the years, CUR has gained much attention for its widely reported anticancer effect. CUR is isolated from *Curcuma longa* (turmeric), a spice native to India. It has been shown to be therapeutically effective against many human conditions, owing to its anti-inflammatory, anti-oxidant, antibacterial, anticancer, wound healing properties, to name a few (Krausz et al., 2015; Vallianou et al., 2015). However, clinical use of CUR is often restricted due to its low water solubility, resulting in poor absorption following oral administration (Anand et al., 2007). It is also rapidly metabolized by the liver and excreted in the feces (Metzler et al., 2013). These unfavorable characteristics have caused CUR to have a very low bioavailability, resulting in sub-therapeutic blood concentration. Therefore, CUR nanoformulations are developed to improve curcumin delivery, thereby overcoming the low therapeutic effects (Torchilin, 2009; Lee et al., 2014). Over the past decades, various nanotechnology-based systems, such as liposomes, micelles, polymeric nanoparticles, nanogels, dendrimers, nanoemulsion, cyclodextrin complexes, solid lipid nanoparticles (SLN), phytosomes, gold nanoparticles, and magnetic nanoparticles are being explored in the pursuit to improve aqueous solubility and drug delivery to the pathological site (Bose et al., 2015; Yallapu et al., 2015).

This review focuses on the design and development of various CUR nanoformulations with special emphasis on CRC therapy. The key properties of CUR, pharmacokinetics and efficacy of CUR nanoformulations in CRC conducted *in vitro* and *in vivo* are discussed, as well as clinical trials of CUR nanoformulations on CRC.

## Background of Colorectal Cancer

Cancer remains as one of the leading causes of death worldwide that is responsible for up to 9.6 million deaths in 2018, resulting in ~1 in 6 deaths (WHO, 2018). Based on the data from 2013 to 2015, the lifetime risk of an individual developing cancer is ~ 4.2%. CRC is responsible for 8.1% of all newly diagnosed cancer cases, and 8.3% of all cancer deaths in 2018. The 5 year survival rate of a patient after being diagnosed with CRC is 64.5% (NIH, 2018). The common risk factors for CRC are non-modifiable factors such as age and genetic factors. The risk of developing CRC increases after 40 years of age, and more than 90% of CRC cases were diagnosed in patients older than 50 years old. Family history of CRC or adenomatous polyps accounts for up to 20% of individuals with CRC. Furthermore, inherited genetic conditions such as familial adenomatous polyposis (FAP) and hereditary non-polyposis colorectal cancer (HNPCC) are responsible for about 5 to 10% of CRC. Genetic mutations are especially notable in these inherited conditions, where mutations in the tumor suppressor gene APC take place in FAP, and mutations in the MLH1 and MSH2 genes in the DNA repair pathway are observed in HNPCC (Haggar and Boushey, 2009). The most common tumor location in CRC is in the proximal colon, followed by rectum and distal colon. Different tumor sites in CRC have different clinical and biological presentations, prognosis, as well as response to treatment (Siegel et al., 2017).

CRC usually begins as a polyp, which is a localized growth on the inner lining of the colon or rectum. Polyps with malignant features have the potential to progress to cancer, though not all polyps evolve to be invasive cancer. Adenomas are polyps with malignant potential, accountable for about 96% of CRC (American Cancer Society, 2017). Over time, the size of polyp increases due to proliferation of cells. This causes genetic mutations and epigenetic changes, therefore the polyp continues to invade nearby tissues and protrude into the bowel wall. As the tumor continues to grow, it becomes more vascularized and eventually the cancerous cells spread to distant metastatic sites through the lymph and circulatory systems (Simon, 2016; American Cancer Society, 2017). Thus, screening and early detection of pre-cancerous polyps are crucial in preventing the progression of polyp to cancer and reducing the incidence rate of CRC. The American Cancer Society recommends that individuals with average risk of CRC should undergo screening at the beginning of 50 years of age. Individuals with increased risk may start screening earlier, before the age of 50. CRC screening includes visual examinations and stool-based tests where a positive result warrants a colonoscopy for diagnosis of CRC (American Cancer Society, 2017).

## Key Properties of Curcumin

**Figure 1:**
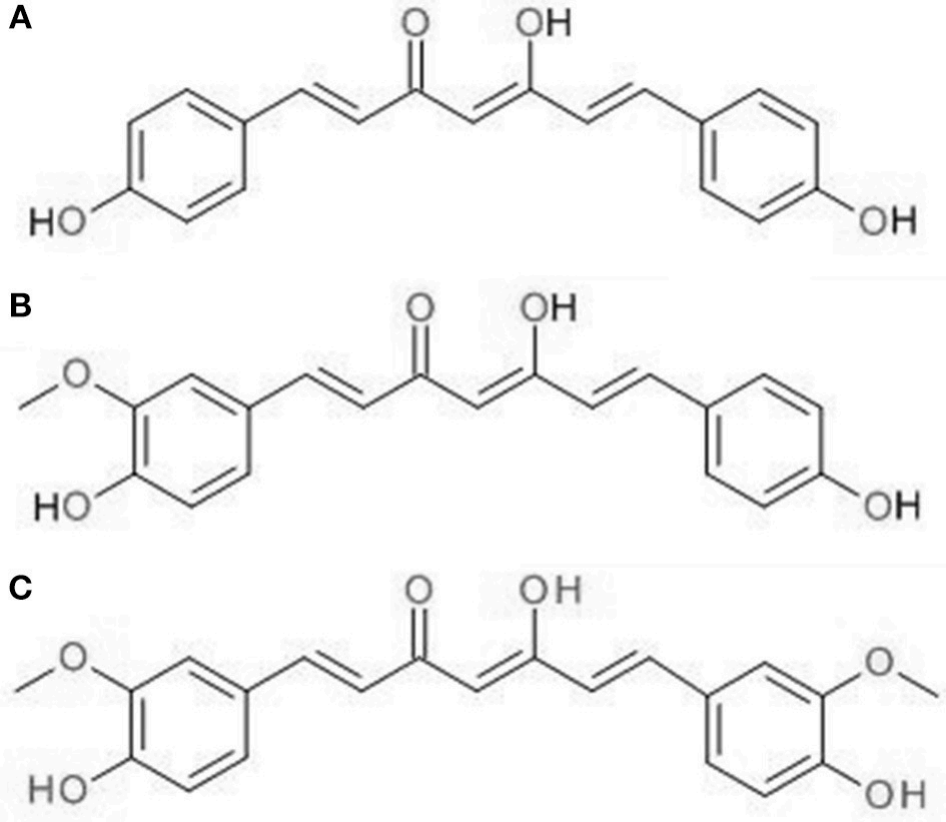
Chemical structures of **(A)** CUR, **(B)** demethoxycurcumin, **(C)** bis-demethoxycurcumin.

CUR is the active constituent of *C. longa* (turmeric), a native Indian spice. In Asian countries, it has a long history of use against many human ailments and skin conditions including acne and psoriasis (Sandur et al., 2007). Besides that, it is also a coloring agent and food additive. It is a crystalline compound with bright orange-yellow appearance, contributing to its use as a coloring agent. Commercial CUR products contain a mixture of curcuminoids, including ~77% CUR, 17% demethoxycurcumin, and 3% bis-demethoxycurcumin. Figure 1 shows the chemical structures of the three curcuminoids. Sandur et al. showed that curcuminoids have different potencies in suppressing the activation of tumor necrosis factor (TNF)-induced nuclear factor-κB (NF-κB). This is most likely due to the differences in the number of methoxy groups found on the ortho position of phenyl ring. At 25 μM, CUR has been shown to be the most potent among the three curcuminoids, followed by demethoxycurcumin and bis-demethoxycurcumin at equivalent concentration (Sandur et al., 2007).

### Physicochemical Properties

The chemical formula of CUR is C_21_H_20_O_6_, with IUPAC nomenclature of [1,7-bis(4-hydroxy-3-methoxyphenyl)-1,6-heptadiene-3,5-dione] and molecular weight of 368.38 Da (Priyadarsini, 2014). CUR displays keto-enol tautomerism with two isomer forms, enol form, and β-diketo form. The predominant isomer depends on the nature of solvent. In acidic and neutral solution, the keto form will predominate; while in alkaline medium, the stable enol form will predominate (Shen and Ji, 2007; Liu et al., 2013). The enol form is more stable when compared to diketo form by 7.75 kcal/mol, due to the presence of strong internal hydrogen bond and extended conjugation along the molecule in the stable enol form (Shen and Ji, 2007). CUR has a log *P* value of 2.5, allowing it to diffuse readily across cellular membranes (Fujisawa et al., 2004). CUR is soluble in organic solvents such as dimethylsulfoxide, methanol, ethanol and chloroform, but insoluble in water. CUR exhibits two strong absorption bands: one between 410 and 430 nm in the visible region; and another at 265 nm in the ultraviolet region. The three pKa values of CUR were reported to be 7.8, 8.5, and 9.0, attributed by the dissociation of enolic proton and two phenolic protons in CUR (Tønnesen et al., 2002). The estimation of pKa values is based on Nuclear Magnetic Resonance (NMR) and absorption spectrometry. However, it is still debatable whether to assign the lowest pKa value to dissociation of enolic proton or phenolic proton due to differences in experimental techniques (Priyadarsini, 2014). At physiological pH, CUR undergoes degradation rapidly where autoxidation is the major degradation pathway. The early degradation products identified are responsible for topoisomerase poisoning, suggesting that oxidative transformation is necessary to generate an active compound. Topoisomerase poisoning is important in mediating the anticancer properties of many anticancer drugs in clinical use (Gordon et al., 2015).

### Absorption and Metabolism

Oral administration of CUR is well-tolerated at doses up to 12 g/day in clinical studies. However, it often has poor bioavailability due to the low aqueous solubility, poor absorption, high first-pass metabolism, and rapid excretion. CUR has a very low solubility of 0.6 μg/ml in water and undergoes rapid degradation in alkaline environment (Naksuriya et al., 2014). The low solubility of CUR leads to poor absorption from the digestive tract following administration through oral route. Early studies performed in rats have shown that about 75% of CUR was eliminated in the feces after oral administration of a 1 g/kg dose of CUR (Wahlström and Blennow, 1978). Similarly, a rodent study conducted by Yang et al. showed a maximum plasma concentration of 0.06 ± 0.01 μg/ml after administration of 500 mg/kg CUR orally (Yang et al., 2007). In a clinical study using human volunteers, CUR serum concentration 1 h post-administration of 2 g CUR orally was either undetectable or very low (< 10 ng/ml) (Shoba et al., 1998). All of these studies have concluded that the serum concentration of CUR was only found in the nano-molar range after more than 1 g of oral CUR doses, indicating the low oral bioavailability of CUR. CUR is mainly metabolized in the liver, and to a lesser extent, in the intestine where glucuronidation is the predominating metabolism pathway. Among the metabolites detected in plasma include glucuronide and sulfate conjugates, with the major metabolite being the glucuronide of hexahydrocurcumin (Asai and Miyazawa, 2000; Ireson et al., 2001; Vareed et al., 2008). In a clinical study, co-administration of 2 g CUR and 20 mg piperine appeared to cause a remarkable 2,000% increase in oral bioavailability. It was found that piperine has the ability to alter the metabolism of CUR by blocking the glucuronidation process in the liver and intestine, thereby improving the CUR oral bioavailability significantly (Shoba et al., 1998).

### Anticancer Properties

**Figure 2:**
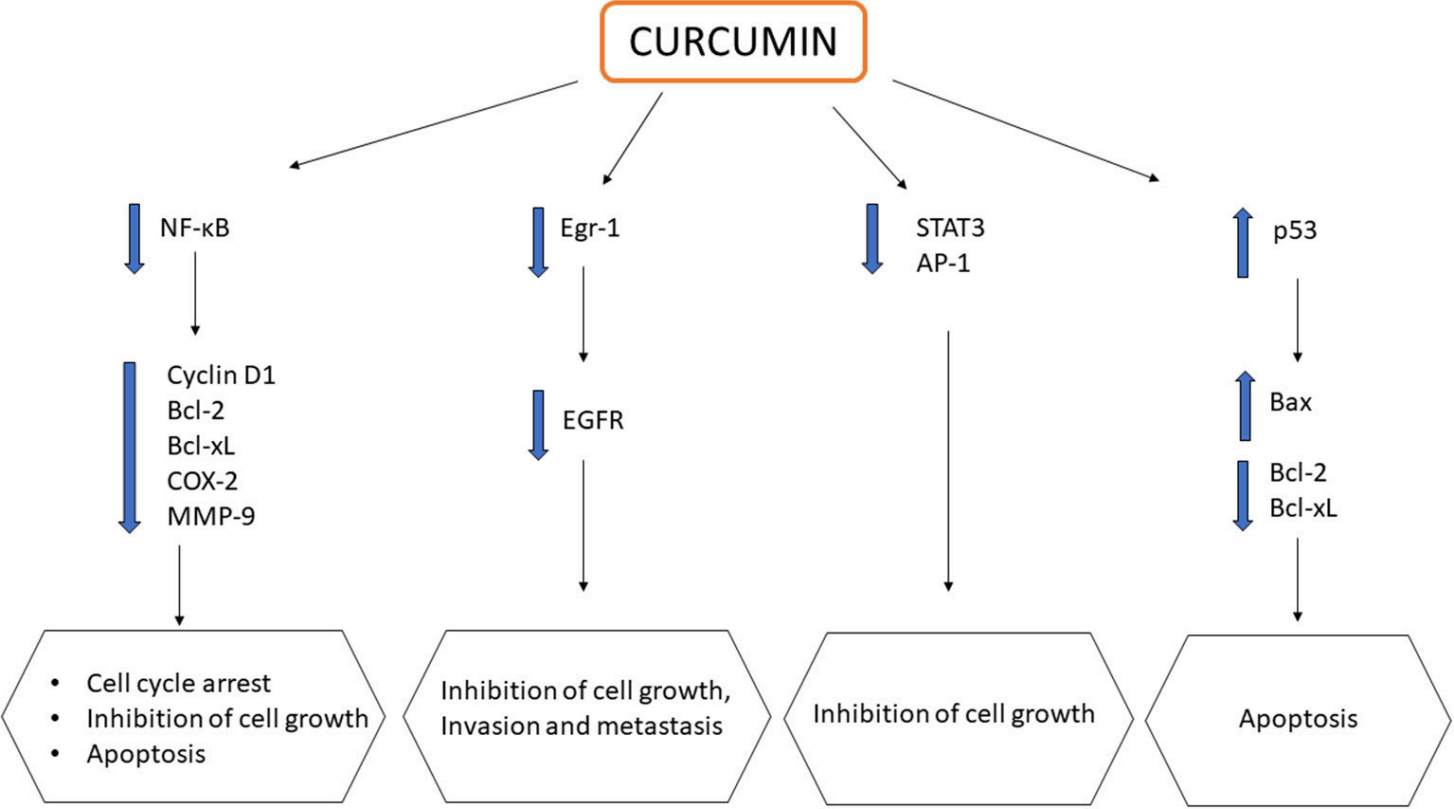
Schematic representation showing impact of curcumin on multiple signaling pathways in cancers.

CUR, alone or in combination, displays chemopreventive and anticancer activities with reported uses against various cancers, including colorectal (Shehzad et al., 2013), pancreatic (Ma et al., 2014), breast (Lv et al., 2014), prostate (Guo et al., 2013), lung (Jin et al., 2015), and oral cancers (Zhen et al., 2014). CUR exerts its effects by downregulating multiple cell signaling pathways, which include NF-κB, STAT3, activated protein-1 (AP-1) and epidermal growth response-1 (Egr-1), which are all crucial in the development and progression of cancer. These transcription factors are usually upregulated in most cancers to assist in cell proliferation, angiogenesis, and the formation of tumors. The downregulation of NF-κB pathways modulates the expression of a variety of genes such as cyclin D1, Bcl-2, Bcl-xL, cyclooxygenase 2 (COX-2), matrix metalloproteinase (MMP)-9, thereby promoting cell cycle arrest, suppressing cell proliferation, and inducing apoptosis (Zhou et al., 2011; Kunnumakkara et al., 2017). CUR also modulates both AP-1 and STAT3 in cellular proliferation of cancer cells. The downregulation of both AP-1 and STAT3 results in retardation of the growth of cancer cells. Epidermal growth factor receptor (EGFR) is a crucial target in cancer treatment as it can be found in many solid tumors including CRC. The downregulation of EGFR is associated with the inhibition of cancer cell growth, invasion and metastasis (Kunnumakkara et al., 2017). CUR was found to suppress the expression of EGFR, mediated by the reduction of Egr-1 activity in Caco2 and HT29 colon cancer cells, inhibiting colon cancer cell growth (Chen et al., 2006). Furthermore, CUR could also promote apoptosis via the upregulation of tumor suppressor gene p53, causing cell death at G2 phase. The downstream effect of p53 activation leads to a net effect of apoptosis in colon carcinoma cells through the downregulation of anti-apoptotic genes Bcl-2/Bcl-xL and upregulation of pro-apoptotic genes Bax (Sa and Das, 2008). The effects of CUR on multiple signaling pathways are summarized in Figure 2. Despite having the above mentioned anticancer properties, its poor oral bioavailability still remains as a main drawback, limiting its clinical potential (Anand et al., 2007). As a result, these issues have led to the development of CUR nanoformulations in the quest of improving CUR delivery for better therapeutic outcome.

## Curcumin Nanoformulations in Colorectal Cancer

**Figure 3:**
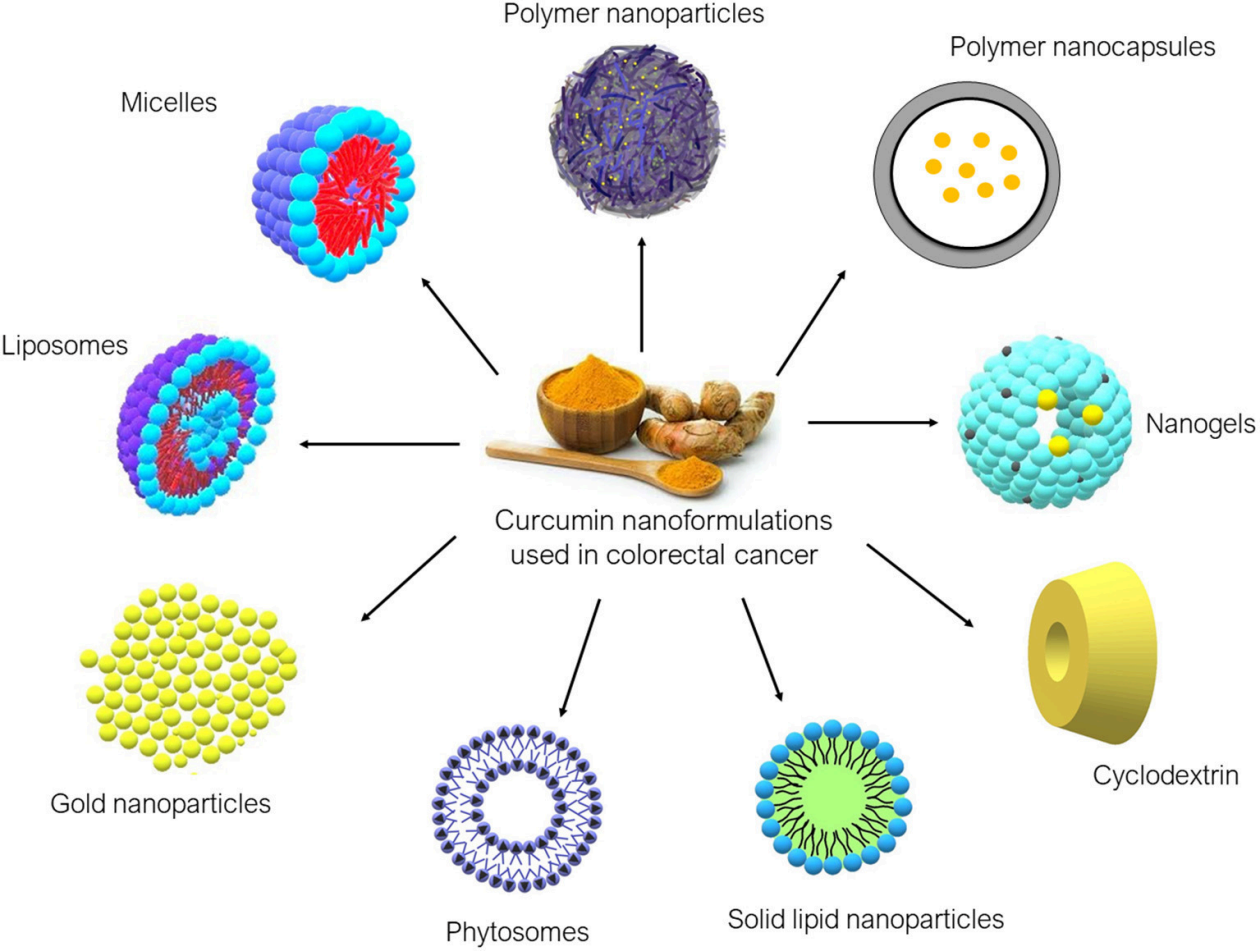
Curcumin nanoformulations used in colorectal cancer found in the literature.

Over the years, various nanoformulations were being explored to enhance CUR delivery to tumor sites. Nanoformulations are primarily used to improve water solubility and to provide a more stable delivery system for CUR. Ideally, CUR nanoformulation for cancer should have enhanced anticancer activity compared to free CUR, at the same time being non-toxic to normal cells. CUR nanoformulations for CRC that have been reported in the literature include liposomes, micelles, polymeric nanoparticles, nanogels, cyclodextrin, SLN, phytosomes, and gold nanoparticles. The diagrams of these nanoformulations are shown in Figure 3, and the findings from the literature are summarized in Table 1.

**Table 1:** Summary of articles on CUR nanoformulations used in colorectal cancer.

**Title**	**Year**	**Type of nanoformulation**	**Outcomes**		**References**
			***In vitro***	***In vivo*/*Ex vivo***	
Liposomal curcumin with and without oxaliplatin: effects on cell growth, apoptosis, and angiogenesis in colorectal cancer	2007	Liposomes	Pegylated liposomal CUR demonstrated a better growth inhibitory effect in Lovo cells than oxaliplatin and demonstrates equivalent growth inhibition in Colo205 cells. Liposomal CUR with oxaliplatin with a ratio of 4:1 showed synergistic effect.	Liposomal CUR reduced tumor growth and displayed antiangiogenic effect in Colo205 and Lovo xenografts. No synergy was observed in liposomal CUR with oxaliplatin.	Li et al., 2007
Preparation and characterization of lyophilized egg PC liposomes incorporating curcumin and evaluation of its activity against colorectal cancer cell lines.	2011	Liposomes	Liposomal CUR provided a more stable delivery of CUR and superior cytotoxic activity to free CUR in long-term assay against HCT116 and HCT15 cell lines.	–	Pandelidou et al., 2011
Native and beta-cyclodextrin-enclosed curcumin: entrapment within liposomes and their *in vitro* cytotoxicity in lung and colon cancer	2012	Cyclodextrin entrapped within liposomes	βCD-C complexes when entrapped within liposomes retained the anticancer activity of liposomal CUR in SW-620 colon cancer cell lines but caused an increase in EC50 value despite a greater water solubility than free CUR.	–	Rahman et al., 2012
Development of antiproliferative long-circulating liposomes coencapsulating doxorubicin and curcumin, through the use of a quality-by design approach	2017	Liposomes	Co-encapsulation of CUR and DOX in LCL caused a remarkable reduction inC26 murine colon cancer cell proliferation compared to free doxorubicin, demonstrating an enhancement of cytotoxic activity.	–	Tefas et al., 2017
Anti-angiogenic and anti-inflammatory effects of long-circulating liposomes co-encapsulating curcumin and doxorubicin on C26 murine colon cancer cells	2018	Liposomes	Co-delivery of CUR and DOX in LCL showed stronger inhibition on cell proliferation on C26 cells than free CUR-DOX as well as free CUR and DOX alone.	–	Sesarman et al., 2018
A W/O emulsion mediated film dispersion method for curcumin encapsulated pH-sensitive liposomes in the colon tumor treatment	2018	Liposomes	CUR-loaded CaCO3 encapsulated liposomes (LCL) showed a greater anti-proliferation effects than liposomal CUR and free CUR in HCT116 cells.	LCL demonstrated the highest reduction in tumor volume of colon cancer model compared to other CUR preparations.	Chen et al., 2018
Curcumin-loaded biodegradable polymeric micelles for colon cancer therapy *in vitro* and *in vivo*.	2011	Micelles	C-26 colon carcinoma cells treated with CUR/MPEG-PCL micelles had a slightly lower cytotoxicity, indicating the slow release of CUR.	CUR/MPEG-PCL micelles prevented subcutaneous growth of C-26 colon carcinoma in mice and improves the anticancer activity of free CUR.	Gou et al., 2011
Novel micelle formulation of curcumin for enhancing antitumor activity and inhibiting colorectal cancer stem cells.	2012	Micelles	CUR loaded CSO-SA micelles increased the cellular uptake and displayed 6-fold higher cytotoxic activity than free CUR in primary CRC cells.	CUR-loaded CSO-SA micelles reduced the size of tumor and the subpopulation of CD44+/CD24+ cell in nude mice tumor tissue.	Wang et al., 2012
*In vitro* cytotoxicity and cellular uptake of curcumin-loaded Pluronic/Polycaprolactone micelles in colorectal adenocarcinoma cells.	2013	Micelles	CUR-loaded Pluronic/PCL micelles improved the cellular uptake of Caco2 colorectal adenocarcinoma cells as well as *in vitro* cytotoxicity due to enhanced aqueous solubility of CUR.	–	Raveendran et al., 2013
Anti-cancer activity of anti-GLUT1 antibody-targeted polymeric micelles co-loaded with curcumin and doxorubicin.	2013	Micelles	CUR+DOX-loaded micelles with the attachment of anti-GLUT1 antibody demonstrated strong cytotoxicity activity compared to the non-targeted formulation in HCT116 cell line.	Both GLUT1-targeted CUR loaded micelles and CUR+DOX-loaded micelles significantly inhibited the growth of tumor and improved the rate of survival of nude mice with HCT116 tumors.	Abouzeid et al., 2013
Curcumin-encapsulated polymeric micelles suppress the development of colon cancer *in vitro* and *in vivo*.	2015	Micelles	CUR micelles demonstrated slower release, increased cellular uptake and increased apoptosis induction but only showed minor enhancement in cytotoxic activity against CT26 colon carcinoma cells	CUR micelles inhibited subcutaneous tumor growth of CT26 colon.	Yang et al., 2015
Curcumin-loading-dependent stability of PEGMEMA-based micelles affects endocytosis and exocytosis in colon carcinoma cells.	2016	Micelles	CUR-loaded micelles showed a greater internalization than unloaded micelles. Smaller CUR-loaded micelles showed a remarkable reduction in WiDr cell proliferation when compared with unloaded micelles and free CUR.	–	Chang et al., 2016
Curcumin mediated down-regulation of αvβ3 integrin and up-regulation of pyruvate dehydrogenase kinase 4 (PDK4) in Erlotinib resistant SW480 colon cancer cells	2018	Micelles	CUR-loaded and erlotinib-loaded MPEG-PCL micelles displayed a synergistic effect by causing increased expression of PDKS and decreased expression of αvβ3 integrin.	–	Javadi et al., 2018
Cellular uptake and anticancer effects of mucoadhesive curcumin-containing chitosan nanoparticles.	2014	Polymeric nanoparticles	CUR-CS-NP showed greater CUR uptake and better anticancer effects in HT29 cells than free CUR due to better mucoadhesive properties of the formulation.	–	Chuah et al., 2014a
Epithelial cell adhesion molecule aptamer functionalized PLGA-lecithin-curcumin-PEG nanoparticles for targeted drug delivery to human colorectal adenocarcinoma cells	2014	Polymeric nanoparticles	Apt-CUR nanoparticles demonstrated superior antiproliferation activity in HT29 colon cells than free CUR at the same concentration. It also showed a stronger cytotoxic effect in EpCAM+ HT29 cells compared to EpCAM- HEK293T.	CUR nanoparticles enhance the bioavailability of free CUR as noted by the prolonged half-life in the pharmacokinetics study performed in rat models.	Li et al., 2014
*In vitro* combinatorial anticancer effects of 5-fluorouracil and curcumin loaded N,O-carboxymethyl chitosan nanoparticles toward colon cancer and *in vivo* pharmacokinetic studies.	2014	Polymeric nanoparticles	The combination treatment of 5-FU and CUR loaded in N,O-CMC NPs in HT29 cells show an enhancement of anticancer effect.	*In vivo* studies using Swiss Albino mice showed that combination treatment of 5-FU and CUR loaded in N,O-CMC NPs displayed an improvement in plasma concentration by extending the plasma half-life compared with the free drug.	Anitha et al., 2014
Co-delivery of camptothecin and curcumin by cationic polymeric nanoparticles for synergistic colon cancer combination chemotherapy.	2015	Polymeric nanoparticles	The combination treatment of CPT and CUR at a ratio of 4:1 loaded in chitosan-functionalized PLGA nanoparticles showed the most effective synergistic anticancer activity on Colon-26 cells than any individually loaded nanoparticles.	–	Xiao et al., 2015b
Hyaluronic acid-functionalized polymeric nanoparticles for colon cancer-targeted combination chemotherapy.	2015	Polymeric nanoparticles	CPT/CUR hyaluronic acid-functionalized nanoparticles with a 1:1 weight ratio demonstrated the best anticancer activity in Colon-26 cells than any individually loaded nanoparticles.	*Ex vivo* study revealed that HA-CUR-NPs were able to penetrate and accumulate in the colon tumor tissues but not at the adjacent or healthy colon tissues.	Xiao et al., 2015a
Curcumin-loaded polymeric nanoparticles for enhanced anti-colorectal cancer applications.	2015	Polymeric nanoparticles	CUR-loaded chitosan-gum arabic nanoparticles displayed superior anti-CRC activity against HCT116 and HT29 cells.	–	Udompornmongkol and Chiang, 2015
Curcumin-polymeric nanoparticles against colon-26 tumor-bearing mice: cytotoxicity, pharmacokinetic and anticancer efficacy studies	2016	Polymeric nanoparticles	CUR-loaded Eudragit^®;^ E100 particles (CENPs) showed a higher cell growth inhibition in Colon-26 cells than CUR aloneinding and cellular uptake of polymeric nanoparticles thus improving cytotoxic activity. CUR-loaded polymeric nanoparticles also reported a greater suppression of tumor growth in tumor-bearing mice after 30 days of administration	CNEPs greatly suppressed tumor growth in Colon-26 tumor-bearing mice after 30 days of treatment with daily dose of 50 mg/kg.	Chaurasia et al., 2016
Supercritical carbon dioxide-developed silk fibroin nanoplatform for smart colon cancer therapy	2017	Polymeric nanoparticles	CUR-SF nanoparticles demonstrated better anticancer potential than free CUR and 5-FU at CUR concentration higher than 10 μg/mL after 6 days of treatment in HCT116 cells. It also showed a reduced cytotoxicity activity to normal colon cells (NCM460 cells).	–	Xie et al., 2017
Co-delivery of curcumin and chrysin by polymeric nanoparticles inhibit synergistically growth and hTERT gene expression in human colorectal cancer cells	2017	Polymeric nanoparticles	PEGylated PLGA nanoparticles containing CUR and Chr showed synergistic cytotoxicity activity evident by the combination indices of < 1. It was also shown to significantly inhibit proliferation of Caco2 cells by further reduction of hTERT expression.	–	Lotfi-Attari et al., 2017
Development and optimization of polymeric self-emulsifying nanocapsules for localized drug delivery design of experiment approach	2014	Polymeric nanocapsules	Polymeric self-emulsifying nanocapsules (PSN) displayed an inhibition of cell proliferation against HT29 cell lines by decreasing IC50 value from 28.56 to 20.32 μM.	In guinea pig model, PSN via oral route showed a low plasma concentration, suggesting limited systemic absorption and reduced clearance.	Wadhwa et al., 2014
Curcumin encapsulated pH sensitive gelatin based interpenetrating polymeric network nanogels for anti cancer drug delivery.	2015	Nanogels	CUR-loaded NGs demonstrated a higher aqueous dispersibility and exhibited excellent anticancer activity *in vitro* toward CRC HCT116 cell line when compared to pure CUR.	–	Madhusudana Rao et al., 2015
Development of curcumin-cyclodextrin/cellulose nanocrystals complexes	2016	Cyclodextrin	CUR-CD/CNCx complexes demonstrated a lower IC50 values and greater antiproliferative effect against HT29 colon cancer cell lines than CUR alone.	–	Ndong Ntoutoume et al., 2016
Chitosan nanoparticles for lipophilic anticancer drug delivery: Development, characterization and *in vitro* studies on HT29 cancer cells.	2016	Cyclodextrin	*In vitro* studies performed on HT29 cells showed no significant difference between both loaded and unloaded nanoparticles with chitosan, hyaluronic acid, sulphobutyl-ether-β-CD with the molar ratio of 20:0:1 in terms of reduction in HT29 cell proliferation treatment due to insufficient CUR concentration (0.5 μM).	–	Abruzzo et al., 2016
Selection and optimization of nano-formulation of P-glycoprotein inhibitor for reversal of doxorubicin resistance in CoLo205 cells	2017	Cyclodextrin	CUR-loaded HP-β-CD with PVA as stabilizer to improve encapsulation of CUR and enhanced the aqueous solubility of CUR. Beyond 40 μM, CUR-loaded HP-β-CD significantly reversed DOX resistance acquired by administration of DOX liposomes with concentration ranging from 0.1 to 10 μM.	–	Dash and Konkimalla, 2017
Formulation of curcumin-loaded solid lipid nanoparticles produced by fatty acids coacervation technique.	2011	Solid lipid nanoparticles	*In vitro* preliminary study conducted on HCT116 colon cancer cells showed no significant difference between CUR-loaded steric acid-SLN and unloaded SLN in terms of cytotoxicity activity.	–	Chirio et al., 2011
Phytosomal curcumin inhibits tumor growth in colitis-associated colorectal cancer	2018	Phytosome	Phytosomal CUR enhanced the anti-proliferation of 5-FU, reduced size of CRC spheroids and reduced cell invasion and migration compared to untreated group in C26 cells. Based on the *in vivo* study in mouse models, CUR/5-FU with dose of 25 mg/kg/day of CUR and 35 mg/kg once weekly of 5-FU significantly suppressed tumor growth via Wnt/β-catenin pathway	Phytosomal CUR and 5-FU combination therapy significantly suppressed tumor growth in colorectal tumorigenesis mouse model.	Marjaneh et al., 2018
Anti-cancer, pharmacokinetics and tumor localization studies of pH-, RF- and thermo-responsive nanoparticles.	2015	Gold nanoparticles	–	The *in vivo* tumor localization studies conducted with mice bearing CT26 mouse colon carcinoma cells showed that Au-CRC-TRC-NPs remained in tumor for up to 2 weeks due to extended blood circulation of CUR. *Ex vivo* imaging in mouse tissues showed a maximum CUR accumulation in tumor compared to other organs where no significant accumulation was observed.	Sanoj Rejinold et al., 2015

### Curcumin Loaded Liposomes

Li et al. assessed both *in vitro* and *in vivo* antitumor activity of CUR encapsulated in liposome for use in CRC. After 72 h of treatment in Lovo cells, it was found that liposomal CUR exhibited a lower IC50 (IC50 = 7.5 μmol/L), demonstrating a greater growth inhibitory effect than oxaliplatin (IC50 ≥40 μmol/L), the standard chemotherapy used for CRC. However, both liposomal CUR and oxaliplatin show equivalent effect in Colo25 cells. In the same study. liposomal CUR was also found to be able to induce apoptosis reflected by PARP cleavage; and prevent angiogenesis by the reduction of angiogenic factors, including CD-31, VEGF and interleukin-8. Similar to *in vitro* studies, liposomal CUR showed significantly greater growth inhibitory effect when compared to oxaliplatin in xenograft models (both Colo25 and LoVo) without overt toxicity. As combination regimen is often employed in cancer therapy to target the different mechanisms on cancer pathway, liposomal CUR was also co-administered with oxaliplatin at a ratio of 4:1. This combination has resulted in a synergistic effect *in vitro*. However, similar effect was not observed *in vivo*. From this study, we can conclude that liposomal CUR has the potential to replace conventional chemotherapeutic agents in CRC, owing to its greater antitumor activity than oxaliplatin. Nevertheless, formal toxicology studies should be performed on liposomal CUR before introduction into clinical setting (Li et al., 2007). Moreover, there has been some inconsistencies in results whereby different cell lines responded differently to the treatments, and the disparities between *in vitro* and *in vivo* results. Hence, careful investigation should be carried out to understand if different genetic make ups would respond differently to the treatment.

In another study, liposomal formulation made of egg phosphatidylcholine (EPC) was used to incorporate CUR, with CUR to lipid molar ratio of 1:14. It was shown that in HCT116 colorectal cells, liposomal CUR exhibited greater uptake rate, suggesting that liposomal CUR provided a more stable delivery of CUR to cells, despite a reduced total intracellular amount of CUR detected *in vitro*. EPC liposomal CUR also has lower IC50 and demonstrated superior cytotoxic activity compared to free CUR in a 2 weeks long-term assay against CRC cell lines (Pandelidou et al., 2011). Thus, it was concluded that incorporating CUR into liposomes can overcome the absorption issues of CUR in the gastrointestinal tract, allowing systemic administration whilst maintaining or even improving its antitumor activity. A recent study performed using C26 murine colon cancer cells revealed that co-encapsulation of CUR and doxorubicin (DOX) in long circulating liposomes (LCL) at a molar ratio of 1:167 caused a remarkable reduction in C26 cell proliferation as compared to free DOX *in vitro*, It was also reported that the extent of the synergistic cytotoxic effects of DOX and CUR depends on the CUR concentration encapsulated; whereby higher CUR amount leads to greater cytotoxic effects (Tefas et al., 2017). Sesarman et al. reported a similar finding when co-delivering CUR and DOX, using the same liposomal formulation as Tefas et al. (LCL-CUR-DOX) in C26 cells. The enhancement in cytotoxic activity of LCL-CUR-DOX over free CUR-DOX encouraged the authors to further investigate the protumor process responsible for the cytotoxicity effects on C26 cells. LCL-CUR-DOX causes a slight inhibition on NF-κB activation, at the same time inhibits majority of proteins involved in tumor development. However, it has less impact on oxidative stress reduction *in vitro* when compared to free CUR-DOX. This could be due to different uptake mechanism of liposomes from free drugs. Liposomes are taken up into the cells via the endocytosis process which enables a greater internalization of CUR. In contrast, free CUR enters the cells via transmembrane diffusion (Sesarman et al., 2018). LCL also differs from conventional liposomes in the sense that the PEGylated surface offers a greater accumulation and a higher CUR concentration at the tumor site through enhanced permeability and retention (EPR) effect, thereby enhancing the antitumor actions.

As previously shown, co-administration of CUR with other chemotherapeutic agents could have synergistic antitumor activity, and this could be a potential solution to the drug resistance and dose-limiting toxicity associated with chemotherapy. Recently, calcium carbonate (CaCO_3_) was investigated in nanoparticles design for its pH sensitive properties to achieve specific targeting of drug release. A new method for the development of liposomes, W/O emulsion mediated film dispersion has proven successful in loading CUR into liposomes and CaCO_3_ encapsulated liposomes (LCC). LCC showed greater cytotoxicity than liposomal CUR and free CUR in HCT116 cells. This was followed by an *in vivo* study on colon cancer model which revealed the greatest reduction of tumor volume with LCC. This is attributed to LLC's ability to promote CUR accumulation in response to low pH environment in lysosome, facilitating CUR release in cytosol. However, the pH sensitivity of LCC against lysosome was not studied against other human cell lines *in vitro* and *in vivo*, therefore it could not be concluded that LCC provided specific targeting to tumor tissues, since lysosomes can be found in normal tissues as well (Chen et al., 2018).

On the other hand, Rahman et al. explored the effects of using a combination of nanocarriers in the delivery of CUR. They have formulated β-CD-CUR (βCD-C) inclusion complexes that were entrapped within liposomes. The βCD-C complexes display a much greater water solubility compared to free CUR. The formulation was then subjected to *in vitro* cytotoxicity studies that were conducted using SW-620 colon cancer cell lines. Both βCD and liposomes encapsulation did not reduce the anticancer effects of CUR in inhibiting cell proliferation. However, CUR-loaded liposomes containing βCD-C (3.25 μM) were reported to have EC50 value greater than CUR-loaded liposomal formulations (0.96 μM). A possible explanation for the less effective anticancer effects for liposomes containing βCD-C may be the fact that it is caused by a reduced *in vitro* drug release, which was not investigated in this study (Rahman et al., 2012). Though the entrapment of cyclodextrin within liposomes comes with the combined advantages of both liposomes and cyclodextrin delivery systems, the drug release mechanisms should be investigated and improved for maximum therapeutic effects.

### Curcumin Loaded Micelles

A single-step nano-precipitation method was used to load CUR into monomethoxy poly (ethylene glycol)-poly (ε-caprolactone) (MPEG-PCL) micelles. The encapsulation of CUR in micelles enables it to be completely soluble in water, forming an injectable CUR formulation. After 48 h of exposure, C-26 colon carcinoma cells that were treated with CUR-loaded micelles showed a slightly lower cytotoxicity than free CUR, indicating slow release of CUR *in vitro*. Furthermore, CUR/MPEG-PCL micelles induced angiogenesis inhibition *in vivo* to a greater extent, compared to free CUR in alginate-encapsulated tumor cell assay. In mouse model, CUR/MPEG-PCL micelles treated group showed smaller tumor volumes, reflecting the ability of this CUR formulation to prevent C-26 colon carcinoma subcutaneous growth. This *in vivo* result proved that CUR loaded MPEG-PCL micelles could improve the anticancer activity of CUR by overcoming the problem of low solubility (Gou et al., 2011). Wang et al. have showed that CUR-loaded steric acid-g-chitosan oligosaccharide (CSO-SA) micelles could protect CUR from biotransformation and hydrolysis, thus improving CUR stability. CUR-loaded CSO-SA micelles increased the cellular uptake in CRC cells; it also exhibited about 6-fold higher cytotoxic activity than free CUR at the same concentration *in vitro* in primary CRC cells. After 14 days of treatment, CUR-loaded CSO-SA micelles also caused a reduction in tumor size and the subpopulation of CD44+/CD24+ cell in nude mice tumor tissue. The increased stability of CUR and improved CUR delivery to tumor sites might be the reason for the enhancement of therapeutic effect of CUR. The inhibition of CD44+/CD24+ cell population showed that CUR could be further explored with the ability to eradicate CRC stem cells as well as bulk cancer cells (Wang et al., 2012).

Pluronic/Polycaprolactone (Pluronic/PCL) amphiphilic block copolymer was used to encapsulate CUR where hydrophobic PCL formed the core to improve CUR loading efficiency. Pluronic block copolymers have been previously reported to stabilize nanoparticles, apart from improving cancer targeting and sensitivity of multidrug resistance tumors. The resultant CUR-loaded micelles have improved the cellular uptake of CUR into Caco2 CRC cells as well as *in vitro* cytotoxicity due to enhanced aqueous solubility when compared to free CUR (Raveendran et al., 2013). On the other hand, Abouzeid et al. investigated the anticancer effects of GLUT1-targeted micelles loaded with CUR and DOX. The overexpression of GLUT1 glucose transporter in many cancer types enables cancer targeting with minimal toxicity on normal tissues. The formulated micelles demonstrated strong *in vitro* cytotoxic activity in HCT116 at low doses of DOX in combination with CUR, evidenced through the reduction in IC50 values of CUR. The GLUT1-targeted formulations significantly inhibited the growth of tumors and improved the survival rate of nude mice with HCT116 tumors *in vivo*. The micelles were also found to have increased internalization via GLUT1, resulting in greater tumor inhibition. In contrast, the synergistic effects of CUR and DOX were not demonstrated in this study, perhaps due to suboptimal dose of DOX used. These results should be investigated in detailed at higher doses to assess the synergistic effects of combination regimen (Abouzeid et al., 2013).

Yang et al. have developed CUR-loaded amphiphilic block copolymers micelles using monomethyl poly (ethylene glycol)-poly (ε-caprolactone) (MPEG-PCL) with the incorporation of trimethylene carbonate (TMC) to form [MPEG-P(CL-co-TMC)] micelles. TMC was incorporated to help stabilize micelles by inhibiting the crystallization of PCL. In this study, CUR micelles result in an increased cellular uptake into CT26 cells, causing a cytotoxic effect and increasing apoptosis induction *in vitro*. However, there were no significant differences in terms of cytotoxic activity against CT26 colon carcinoma cells in comparison to free CUR. Nevertheless, CUR micelles treated group has a significantly lower tumor weight than free CUR, reflecting a stronger *in viv*o antitumor activity. Furthermore, the growth of subcutaneous CT26 tumor was inhibited by CUR micelles *in vivo* via the following mechanisms: prevention of tumor cell proliferation and angiogenesis; and promotion of apoptosis. These findings reveal that addition of TMC could improve the micelles stability, making MPEG-P (CL-co-TMC) micelles a suitable nanocarrier for CUR in colon carcinoma (Yang et al., 2015).

Chang et al. also demonstrated that the size of micelles could impact on CUR delivery to WiDr human colon carcinoma cells. Two different chain lengths of amphiphilic block copolymers were made up of hydrophilic poly (ethylene glycol) methyl ether methacrylate (PEGMEMA) and hydrophobic polystyrene (PS) through reversible addition-fragment chain-transfer (RAFT) polymerization. CUR-loaded micelles showed a greater cellular internalization than unloaded micelles, indicating the presence of CUR was able to improve the micelles thermodynamic stability. After 72 h of exposure, smaller CUR-loaded micelles showed a remarkable reduction in WiDr cell proliferation when compared with unloaded micelles and free CUR. The results also showed that larger micelles undergo endocytosis and exocytosis more rapidly than smaller micelles, affecting the uptake and cytotoxicity effect. Hence, the size of micelles is an important aspect that influences the endocytosis and exocytosis kinetics in the formulation of nanomedicine (Chang et al., 2016).

In recent years, the focus of CUR researches in CRC have been shifted to investigate the potential of CUR to reverse chemotherapy cancer resistant cells as a combinatorial agent with chemotherapy. Combination therapy with CUR-loaded and Erlotinib-loaded MPEG-PCL micelles was evaluated in erlotinib-resistant SW480 colon cancer cells, which were treated with different doses of CUR and erlotinib. In this respect, previous studies have demonstrated that upregulation of αvβ3 integrin and downregulation of pyruvate dehydrogenase kinase 4 (PDK4) led to the development of drug resistance. In this study, the combination therapy was shown to increase the expression of PDK4 and decrease the expression of αvβ3 integrin in resistant cells compared to non-resistant cells, suggesting the role of CUR in prevention of drug resistance (Javadi et al., 2018).

### Curcumin Loaded Polymeric Nanoparticles

Chuah and co-workers have formulated CUR-containing chitosan nanoparticles (CUR-CS-NP) to improve the delivery of CUR to the colon via mucoadhesion. It was anticipated that chitosan would adhere to the mucus in colon, prolonging the contact time of CUR with colon. Interestingly, they observed better mucoadhesion properties of the CUR-CS-NP compared to empty chitosan nanoparticles (CS-NP), suggesting the involvement of CUR in the mucoadhesion process (Chuah et al., 2014a). The mucoadhesion process was also confirmed via reduction in zeta potential of the CUR-CS-NP, as well as Atomic Force Microscopy (AFM) and Nanoparticle Tracking Analysis (NTA) (Chuah et al., 2014b). The formulated CUR-CS-NP also showed greater CUR uptake in CRC cells (HT29) than free CUR due to the mucoadhesive properties, which prolonged the exposure of CUR to the cells. Superior anticancer effects were also observed with CUR-CS-NP whereby cell viability was reduced to a greater extent, along with a reduction in IC50. Both CUR and CUR-CS-NP induced cell apoptosis in HT29 cells, causing cell cycle arrest at G_2_/M phase (Chuah et al., 2014a). On the other hand, polymeric nanoparticles functionalized with targeting ligands have been studied to improve the delivery of CUR to target cancerous cells. These CUR-loaded PLGA-lecithin-PEG nanoparticles were prepared via nanoprecipitation method. CUR nanoparticles were functionalized with aptamers (Apt) that bind specifically to epithelial cell adhesion molecule (EpCAM) found on tumor surfaces. The *in vitro* cytotoxicity study of Apt-CUR nanoparticles showed that Apt-CUR nanoparticles have strong antiproliferation activity in HT29 colon cells compared to free CUR at equivalent concentration of 4 μg/mL; and stronger cytotoxic effect in EpCAM+ HT29 cells relative to EpCAM-HEK293T (human embryonic kidney cells). This could be due to the 64-fold increase in binding of Apt-CUR nanoparticles to HT29 cells, therefore increasing CUR internalization. Based on the *in vivo* pharmacokinetics study in rat models, the CUR nanoparticles caused a 6-fold increase of half-life as compared to suspension of free CUR, leading to enhanced bioavailability (Li et al., 2014). Taken together, polymeric nanoparticles can be functionalized to actively target delivery of CUR to CRC cells while sparing the cytotoxicity on normal tissues.

Anitha et al. attempted to improve the effectiveness of 5-fluorouracil (5-FU) by means of co-encapsulation with CUR. CUR was found to work in synergy in improving 5-FU effectiveness by inhibiting the overexpression of COX2 in colon cancer. Combination treatment of CUR and 5-FU loaded in N,O-carboxymethyl chitosan nanoparticles (N,O-CMC NPs) enhanced the anticancer effects against HT29 cells as compared to that of individual treatments. *In vivo* studies in Swiss Albino mice showed an improvement in plasma concentration with an extension in the plasma half-life of up to 72 h, indicating an enhanced bioavailability with NPs encapsulation compared with the free drug (Anitha et al., 2014). However, the clinical efficacy of combination regimen of 5-FU and CUR loaded NPs were not investigated in colon xenografts despite showing compatibility with blood. Additional safety and efficacy profile in colon xenografts models are necessary to translate these findings to a clinical perspective.

Similarly, in another study, Xiao et al. explored *in vitro* cytotoxicity using Colon-26 cells and the synergistic effects of co-delivery of camptothecin (CPT) and CUR loaded in chitosan-functionalized poly(lactic acid/glycolic acid) (PLGA) nanoparticles. Chitosan functionalization was hypothesized to convert the surface of PLGA nanoparticles to positive charge, promoting binding to negatively charged cell surface and improve cellular uptake. Interestingly, cationic CPT-loaded nanoparticles demonstrated a better cellular uptake compared to free CPT and CPT-loaded nanoparticles, suggesting an enhanced cell surface interaction. Cationic CPT/CUR nanoparticles with a 4:1 weight ratio showed the most effective anticancer activity than the individual treatment groups with the greatest reduction in anti-apoptotic Bcl-2 expression and induction of cell apoptosis (Xiao et al., 2015b). The same authors also conducted a similar study where chitosan was replaced with hyaluronic acid (HA). In this study, HA-CPT/CUR nanoparticles with a 1:1 weight ratio demonstrated the best anticancer activity and the greatest reduction of IC50 values compared to HA-CPT nanoparticles, suggesting that CUR enhanced the efficacy of CPT. The presence of HA also enhanced tumor targeting and cellular uptake in comparison with chitosan nanoparticles due to high affinity of HA for the overexpressed CD44 receptors in colon cancer, mediating the endocytosis process. *Ex vivo* study in colon cancer mouse model revealed that HA-functionalized CUR-loaded polymeric nanoparticles (HA-CUR-NPs) were able to penetrate and accumulate at the colon tumor, but not at the adjacent healthy colon tissues (Xiao et al., 2015a). The results from these two studies corroborate each other, where the combination treatment of CPT and CUR loaded in PLGA nanoparticles on Colon-26 cells display higher cytotoxicity *in vitro* than free CPT. This indicates that CPT and CUR provided a synergistic anti-cancer effect against CRC.

In another study, chitosan and gum arabic were used to formulate CUR-loaded nanoparticles to improve CUR encapsulation and loading efficiency. These positively charged nanoparticles displayed superior anti-CRC activity against HCT116 colon and HT29 CRC cells when compared to free CUR, due to the greater cellular uptake of CUR into the negatively charged cancer cells. The authors also reported an increase in cell population in the subG1 phase during cell cycle analysis, indicating that CUR-NPs were able to induce apoptosis more readily than free CUR (Udompornmongkol and Chiang, 2015). Another polymeric nanoparticles were prepared using Eudragit® E100 cationic copolymer to improve the oral bioavailability and anticancer effects of CUR. This nanoparticle formulation showed a 19-fold higher growth inhibition of Colon-26 cells than CUR alone, reflecting better binding and cellular uptake of polymeric nanoparticles, thus improving cytotoxic activity. CUR-loaded polymeric nanoparticles also reported a greater suppression of tumor growth in tumor-bearing mice after 30 days of administration (Chaurasia et al., 2016). These observations are consistent with earlier reports of polymeric nanoparticles on CRC therapy despite different polymers being used.

Silk fibroin (SF), a natural polymer has recently been investigated to replace synthetic polymers in the preparation of polymeric nanoparticles in an attempt to resolve the biocompatibility issues found in synthetic polymers. CUR-SF nanoparticles were expected to provide a controlled release of CUR to colon cancer cells. The study proved that after a 6 days treatment on HCT116 cells, CUR-SF nanoparticles possess better anticancer potential than free CUR at 5 μg/mL. When CUR and 5-FU were used at the same concentrations above 10 μg/mL, CUR-SF showed greater anticancer effects than 5-FU. Furthermore, it exhibited a reduced cytotoxicity to normal human colon mucosal epithelial cells (NCM460 cells) compared to all other treatment groups. These findings suggest that the incorporation of CUR into SF nanoparticles provide a greater CUR cellular uptake into cancer cells and reduced toxicity to normal cells due to its slow release characteristics (Xie et al., 2017). CUR nanoparticles that were prepared using natural polymers showed no toxicity and is compatible to normal cells. Therefore, it has the potential to replace the use of synthetic polymers in the development of polymeric nanoparticles.

Lately, co-administration of natural compounds with established anticancer activity has been explored based on the use of combinational chemotherapy in clinical settings. PEGylated PLGA nanoparticles containing CUR and chrysin (Chr) were prepared to enhance the poor solubility of both CUR and Chr. The synergistic cytotoxic activity of CUR and Chr was proven by median-effect method with combination indices of < 1. CUR-Chr nanoparticles significantly inhibit proliferation of Caco2 cells compared to free CUR-Chr, CUR alone, and Chr alone. The improvement in cytotoxicity seen with combinational nanoformulation is associated with a greater reduction of hTERT expression, an enzyme involved in telomerase activity (Lotfi-Attari et al., 2017). Though synergism of both natural compounds was proven, this study lacks the comparison of its finding against the standard chemotherapy agents used in CRC such as 5-FU or oxaliplatin. A direct comparison of such findings would be useful in further developing the formulation for clinical use.

Polymeric nanocapsules with self-emulsification ability were developed for the delivery of CUR to target colonic sites. The self-emulsifying properties could improve CUR solubility when exposed to alkaline conditions such as the colonic regions. In this study, researchers have observed that the enteric polymer would dissolve at colonic pH (pH7.2), along with a 5-h lag time in CUR release from the polymeric nanocapsules, allowing targeted delivery of CUR to colon. Furthermore, these polymeric nanocapsules exhibited significant cytotoxicity against HT29 cell lines. A reduction in IC50 value was achieved from 28.56 to 20.32 μM when CUR was formulated in polymeric nanocapsules. *In vivo* study was performed in guinea pig model where the formulations were administered orally with same dose (100 mg/kg) of CUR, either in the form of polymeric nanocapsules suspension or pure suspension. This study showed that polymeric nanocapsules had low plasma concentration of 200 ng/mL, indicating limited systemic absorption. Therefore, it had reduced clearance and prolonged elimination of half-life, permitting a localized delivery of CUR (Wadhwa et al., 2014). The limitation of this study is that the *in vivo* study was not conducted using animal models with colon tumors, therefore the *in vivo* cytotoxicity against colon cancer is not justified.

### Curcumin Loaded Nanogels

Nanogels formulation has been used to encapsulate CUR to protect it from degradation and to provide structural stability. In this study, gelatin polymer and acrylamidoglycolic acid (AGA) polymers act as monomers to form interpenetrating polymeric network nanogels (IPN-NGs) through simple emulsion polymerization for the encapsulation of hydrophobic CUR. Notably, CUR-loaded NGs release CUR to a greater extent at pH 7.4 than at pH 1.2 *in vitro*, suggesting that these nanogels exhibit pH sensitive properties. This pH sensitive properties can be explained by the increase in ionic repulsion at higher pH, causing the swelling of nanogels, allowing a greater release of CUR. CUR-loaded NGs exhibited excellent anticancer activity *in vitro* toward HCT116 cell line compared to pure CUR at a dose of 1 mg/ml after 48 h incubation. The greater anticancer activity is attributed to the higher aqueous dispersibility of the NGs, thus increasing the bioavailability of CUR. This unique pH sensitive behavior of IPN-NGs allowed these nanogels to have a better stability profile at physiological pH (pH 7.4), thereby allowing more CUR release as well as providing protection from degradation (Madhusudana Rao et al., 2015).

### Curcumin Loaded Cyclodextrin

In another formulation, CUR-loaded cyclodextrin (CD) was attached to cellulose nanocrystals (CNCx) to form CUR-CD/CNCx nano complexes. These complexes were reported to show the greatest cellular accumulation of CUR, followed by CUR-CD and CUR when applied to HT29 cells, suggesting the involvement of CNCx in improving cellular uptake. These CNCx possess tumor targeting properties which can improve CUR transport into tumor vasculature via the EPR effect. Furthermore, the *in vitro* study showed a lower IC50 value and a 3-fold greater antiproliferative effect than CUR alone. These results reflected the effects of better aqueous solubility of CUR with the attachment of CNCx to nanoformulations, therefore improving cellular uptake (Ndong Ntoutoume et al., 2016). A preliminary study performed on CRC HT29 cells evaluated the effects of CUR-loaded nanoparticles with different molar ratios of chitosan, HA, sulphobutyl-ether-β-CD (Curc/SBE-β-CD) on CUR internalization and cell proliferation. The drug complexation within cyclodextrin increased the water solubility of CUR from 3.72 to 70 μg/mL. Cyclodextrin act as a solubilizing agent, allowing the incorporation of hydrophobic CUR in chitosan nanoparticles. After 4 h of treatment, cellular internalization of CUR was reduced in I407 cells (normal human intestinal epithelial cells) with the treatment of CUR-loaded chitosan, HA and sulphobutyl-ether-β-CD nanoparticles formulated with the molar ratios of 20:0:1. This formulation also showed an optimal cellular uptake into HT29 cells, suggesting a reduced cytotoxic effect on normal epithelial cells. Impressively, *in vitro* studies performed on HT29 cells showed that these nanoparticles significantly reduced cell proliferation compared to untreated group, but there was no significant difference between both loaded and unloaded nanoparticles in terms of reduction in HT29 cell proliferation after 24 h of treatment, due to insufficient CUR concentration (0.5 μM). The authors concluded that the unloaded nanoparticles were able to cause cell cycle arrest and induce apoptosis of HT29 cells. In this case, CUR did not contribute to the decrease of cell proliferation; on the contrary, the nanoparticle formulation was responsible for the anticancer effects (Abruzzo et al., 2016).

Recently, Dash and Konkimalla have loaded CUR into hydroxypropyl-β-cyclodextrin (HP-β-CD) with Polyvinyl alcohol (PVA) as stabilizer to improve encapsulation of CUR. This nano-curcumin formulation has enhanced the aqueous solubility of CUR. It was used in combination therapy to overcome the problem of drug resistance toward DOX due to p-glycoprotein (P-gp) overexpression. After prescreening, CUR was selected over other P-gp inhibitors as combinatorial agent in this study as it caused sensitization of DOX-resistance Colo205 cells at the lowest concentration of 40 μM. Beyond 40 μM, CUR-loaded HP-β-CD significantly reversed DOX resistance acquired by administration of DOX liposomes with concentration ranging from 0.1 to 10 μM (Dash and Konkimalla, 2017).

### Curcumin Loaded Lipid-Based Nanoparticles

To date, only one study was reported in the literature on the use of CUR SLN formulation for colon cancer therapy. CUR was filled into SLN by coacervation based on fatty acid precipitation. SLN was able to enhance CUR stability by providing protection against hydrolytic and oxidative degradation. According to the *in vitro* preliminary study on HCT116 colon cancer cells, both CUR-loaded and unloaded SLN showed similar cytotoxicity activity at 1:100 and 1:200 dilutions, possibly due to low CUR concentration entrapped within the SLN(Chirio et al., 2011). As the result from this study show no benefits of SLN in improving CUR delivery, SLN might not be a suitable carrier for CUR at this point of time.

Phytosome preparation of CUR involves using phospholipids for the complexation of CUR with the goal of enhancing CUR bioavailability. Comparative analysis of phytosomal CUR alone and in combination with 5-FU was investigated in CT26 cells and colitis-associated CRC mouse model. Phytosomal CUR was shown to enhance the anti-proliferation of 5-FU, shrinking the CRC spheroids and reducing cell invasion and migration compared to untreated group in C26 cells. Based on the *in vivo* study in mouse models, CUR/5-FU with 25 mg/kg/day of CUR and 35 mg/kg once weekly of 5-FU significantly suppressed tumor growth via the Wnt/β-catenin pathway (Marjaneh et al., 2018). These are in agreement with the results from other studies that reported CUR enhanced efficacy of current therapy in CRC.

### Curcumin Loaded Gold Nanoparticles

Sanoj Rejinold et al. evaluated the use of gold nanoparticles (Au-NPs) in chitosan-graft-poly(N-vinyl caprolactam) nanoparticles (Au-CRC-TRC-NPs) to develop pH-, radiofrequency- and thermo-responsive nanoparticles. When exposed to radiofrequency pulses, the Au-NPs could induce heat, triggering CUR release according to the lower critical solution temperature (LCST) action imparted by poly(N-vinyl caprolactam). The *in vivo* tumor localization studies conducted in mice bearing CT26 xenografts showed that Au-CRC-TRC-NPs remained in the tumor for up to 2 weeks, showing the high retention capacity of these positively charged nanoparticles. They were also found to have extended the circulation time of CUR in the blood for up to a week, compared to CUR alone, which was completely eliminated after 6 h. In *ex vivo* imaging study using the formulation, maximum CUR accumulation was observed in the tumor compared to other organs. The promising results showed that Au-CRC-TRC-NPs are tumor specific and could potentially provide a targeted colon cancer treatment regimen (Sanoj Rejinold et al., 2015).

## Clinical Trials of CUR Nanoformulations

The successful developments of CUR nanoformulations and their associated positive *in vitro* and *in vivo* outcomes have led to the commencement of several clinical trials involving these formulations. Clinical trials evaluation is crucial in translating preclinical effects into human body, to further validate the feasibility and efficacy of such formulations for human use in a clinical setting. To date, a total of three clinical trials on CUR nanoformulations for use in CRC and colorectal adenomatous polyps have been registered in the clinical trials database ClinicalTrials.gov.

CUR conjugated with plant exosomes are being explored to improve the delivery of CUR to colon tissues and colon tumors. Exosomes can strongly bind to CUR and many other hydrophobic drugs. The intestine cells and immune cells are able to take up these plant-derived exosomes fairly well, facilitating treatment for intestinal diseases. The subjects were divided into three groups: the first group was given tablets containing 3.6 g of CUR conjugated with plant exosomes daily for 7 days; the second group received tablets containing 3.6 g of CUR alone daily for 7 days; the third group did not receive any intervention. The concentration of CUR in normal and cancer tissues were evaluated as the primary outcome while the secondary outcomes measured the safety, immune response and metabolic characteristics of free CUR and CUR loaded exosomes (NCT number: NCT01294072, 2011).

A phase II clinical study reports on CUR phytosome Meriva^©^, a patented CUR formulation that incorporates food-grade lecithin. The study intervention includes Meriva^©^ and Mirtoselect^©^, an anthocynanin mixture. This study aims to investigate the expression of proteins necessary for the formation of colon tumor caused by the treatment, compared to placebo. Patients with colorectal adenomatous polyps were given 1,000 mg of Mirtoselect^©^ and 1,000 mg of Meriva^©^ daily for 28 days. The change in the expression of biomarker β-catenin in adenomatous tissue and normal rectal mucosa were compared as primary outcome. The immunohistochemical expression of NF-Kβ, Ki-67, and p53 were measured as secondary outcomes (NCT number: NCT01948661, 2013).

Another phase II clinical trial investigates nanostructured lipid CUR particles in the form of a dietary supplement in addition to standard chemotherapy treatment. The recruited subjects are patients with unresectable metastases of CRC. Avastin with folinic acid, fluorouracil and irinotecan (FOLFIRI) chemotherapy are given as the treatment every 14 days. Oral supplement of CUR is given at a dose of 100 mg twice daily until the completion of chemotherapy. The nanostructured lipid CUR particles are expected to increase CUR bioavailability. Subjects will be monitored for 2 years for progression-free survival after the intervention. The overall survival rate and response rate, safety, quality of life, and fatigue scale will also be assessed as the secondary outcomes (NCT number: NCT02439385, 2015).

## Research Gap and Future Directions

Most of the CUR nanoformulations reports found in the literature described the efforts to improve curcumin delivery to overcome the issues of low solubility, poor absorption, rapid metabolism, and limited bioavailability. These were mainly achieved by ways of controlled release, mucoadhesion, targeted delivery, improved stability, and increased cellular uptake. While the majority of these literature showed positive results with enhanced anticancer activities toward CRC, some nanoformulations did not demonstrate superior efficacy than the use of CUR alone. Therefore, it cannot be concluded that the use of all CUR nanoformulations would lead to enhanced anticancer effects toward CRC, rather the effects are specific to certain nanoformulations. Moreover, the efficacy of such formulations also greatly depends on the dose of CUR used. Studies that revealed opposing results warrant further investigations to identify any inconsistencies and address the differences in results. There also appeared to be some limitations in the reported works, such as the absence of *in vivo* studies. It is understandable that there could be time and budget constraints when carrying out the studies. Given that *in vitro* results do not always give rise to similar results *in vivo*, further testing is crucial to make sure that the formulations work as well in an *in vivo* condition, before proceeding to human studies. The latest advances on 3D cell culture model was proposed to be able to mimic the *in vivo* tumor features more closely, and has recently been tested for CUR and CUR emulsions in HCT116 cells (Low et al., 2019; Tan et al., 2019). The results from these studies look promising, though the correlation between the 3D *in vitro* cell culture and *in vivo* data has yet to be established. The pharmacokinetics and biodistribution of these formulations also remain largely unexplored. Tracking of nanoformulations *in vivo* using imaging techniques has been proven to be able to generate useful information regarding the stability, accumulation, and biodistribution of the nanoformulations. Furthermore, there is also a lack of information on the toxicity and biocompatibility of these formulations. These are essential components in the development of a formulation for human consumption. Hence, the gap in this area must be bridged to bring the research on CUR nanoformulations forward.

It is also noteworthy that from the literature, different cell lines responded to treatments differently, possibly due to different characteristics or genetic make ups exhibited by the cells. In relation to patient treatment, it is not surprising that different patients would respond differently to the same treatment. As cancer cells are constantly evolving, a patient could possibly have a clinical presentation with mixed characteristics that would respond to different anticancer agents. In this respect, combination therapy offers a good option as different compounds can target different molecular pathways in halting the progression of cancer. This is especially useful in overcoming chemoresistance toward conventional cancer drugs. Co-administration of curcumin with conventional chemotherapeutic agents have been shown to have synergy effects, which is a promising approach as it also allows the use of lower doses of chemotherapeutic agents. This will inevitably reduce the side effects associated with such treatment. On the other hand, co-encapsulation of two or more natural compounds with anticancer activities is proven useful as well, with the benefit of eliminating the use of toxic chemotherapy completely.

Recently, there has been a lot of debates on the clinical efficacy of CUR. CUR has been classified as both a PAINS (pan-assay interference compounds) and an IMPS (invalid metabolic panaceas) candidate by some researchers (Baell and Walters, 2014; Nelson et al., 2017). It was claimed that these compounds interfere with assays and demonstrate non-drug like mechanisms. The positive results from *in vitro* and *in vivo* studies on curcumin were not seen in clinical trials, where more than 120 clinical trials of curcuminoids against several diseases have failed (Nelson et al., 2017). To date, all the clinical trials on the use of CUR nanoformulations for CRC did not report the outcome, possibly due to on-going investigation or discontinuation of the studies, which is still unknown at the time of writing. Despite the negative remarks, it is no doubt that CUR is safe and well-tolerated in patients, and its benefits most obvious when used in combination with other compounds (Ferri et al., 2018). In fact, the failure of CUR in clinical studies shall not be blamed solely on the likely false activities as claimed by some researchers. It is well-known that CUR has very poor solubility and bioavailability. Without proper formulation, oral administration of CUR indeed has very limited benefit. Careful choice of formulation is desirable to ensure the pharmacological effects of CUR in human studies. Moreover, the source of curcumin also plays an important role, as the purity of the compound greatly affects its activity. Therefore, it is important that CUR be derived from trustable sources with tested purity. Furthermore, to avoid the likely claim of CUR interfering with assays, multiple assays should be carried out to make sure consistent positive results are obtained. It is also vital to validate its mechanism of action and investigate the exact binding site on the proteins for more accurate and convincing data.

With all the research efforts, positive outcomes from such studies would definitely push the development of CUR nanoformulations for CRC another step forward, for the benefit of patients. Before this can happen, the investigations of CUR efficacy should be validated. Thorough characterization of the CUR nanoformulations should be carried out; along with detailed *in vitro* and *in vivo* evaluation; as well as biocompatibility and toxicity assessments. With a strategic research direction, formulation scientists can work toward developing safe and effective CUR nanoformulations for the treatment of CRC.

## Conclusion

In pre-clinical studies, most of the CUR nanoformulations in CRC therapy have been proven successful in enhancing the aqueous solubility, delivery and efficacy over conventional delivery using free CUR except for SLN and conflicting results were seen with cyclodextrin formulation. A large pool of literature on CUR nanoformulations for CRC was conducted *in vitro* using colon and CRC cell lines. However, very limited studies were performed *in vivo* to ascertain the toxicity and therapeutic effect of CUR in animal models to allow translation of these preclinical findings to a clinical perspective. Despite a few reported clinical studies in CRC therapy, more extensive studies and investigations are necessary to prove an increased bioavailability via the encapsulation of CUR into nanoformulations. The complete mechanism as well as the interactions between these nanoformulations and cancer cells are still unknown. Future studies should focus on understanding the effects of CUR nanoformulations on colon and CRC cells through long term studies, as well as CUR interactions with both normal and cancer cells. These are essential for the development of a safer and more effective chemotherapeutic agent taken from bench to bedside.

## Author Contributions

The paper was conceptualized by L-HC and written by KW. L-HC, K-GC, SN, B-HG, and L-HL provided vital guidance and insight into the work.

### Conflict of Interest Statement

The authors declare that the research was conducted in the absence of any commercial or financial relationships that could be construed as a potential conflict of interest.
